# Correction: Receptor tyrosine kinases Tyro3, Axl, and Mertk differentially contribute to antibody-induced arthritis

**DOI:** 10.1186/s12964-023-01293-z

**Published:** 2023-08-31

**Authors:** Liang Gao, Chao He, Aizhen Yang, Haibin Zhou, Qingxian Lu, Raymond B. Birge, Yi Wu

**Affiliations:** 1 Collaborative Innovation Center of Hematology, State Key Laboratory of Radiation Medicine and Prevention, National Clinical Research Center for Hematologic Diseases, Cyrus Tang Medical Institute, Soochow University, Suzhou, 215123 China; 2 Department of Orthopaedics, The Second Affiliated Hospital of Soochow University, Suzhou, 215123 China; 3 Department of Ophthalmology and Visual Sciences, University of Louisville, Louisville, KY 40202 USA; 4 Department of Microbiology, Biochemistry and Molecular Genetics, New Jersey Medical School Cancer Center, Rutgers University, Newark, NJ USA; 5 Sol Sherry Thrombosis Research Center, Temple University School of Medicine, Philadelphia, PA USA

**Correction: Cell Commun Signal 21, 195 (2023)**

**https://doi.org/10.1186/s12964-023-01133-0**

Following publication of the original article [1], the authors identified an error in the author name of Haibin Zhou.

The incorrect author name is: Haibing Zhou

The correct author name is: Haibin Zhou

The author group has been updated above and the original article [1] has been corrected.
