# Supplementary material for: Receptor tyrosine kinases Tyro3, Axl, and Mertk differentially contribute to antibody-induced arthritis
Source: Cell Commun Signal. 2023 Aug 3;21:195. doi: 10.1186/s12964-023-01133-0 (PMC10398921; doi:10.1186/s12964-023-01133-0)
Supplement: Supplementary file 2 — Additional file 1. [file 12964_2023_1133_MOESM1_ESM.docx]

**Receptor tyrosine kinases Tyro3, Axl, and Mertk differentially contribute to**

# antibody-induced arthritis

Liang Gao^1*^, Chao He^1*^, Aizhen Yang^1*^, Haibing Zhou^2^, Qingxian Lu^3^，Raymond B.

Birge^4^, Yi Wu^1,5^

^1^National Clinical Research Center for Hematologic Diseases, Cyrus Tang Medical Institute, Collaborative Innovation Center of Hematology, State Key Laboratory of Radiation Medicine and Prevention, Soochow University, Suzhou 215123, China;

^2^Department of Orthopaedics, The Second Affiliated Hospital of Soochow University, Suzhou 215123, China; ^3^Department of Ophthalmology and Visual Sciences, University of Louisville, Louisville, KY, 40202, USA; ^4^Department of Microbiology, Biochemistry and Molecular Genetics, New Jersey Medical School Cancer Center, Rutgers University, Newark, NJ, USA; ^5^Sol Sherry Thrombosis Research Center, Temple University School of Medicine, Philadelphia, PA, USA.

*These authors made equal contribution to this study.

Corresponding author: Aizhen Yang, PhD, Cyrus Tang Medical Institute, Soochow University, Suzhou 215123, China, Email: yangaizhen@suda.edu.cn; Raymond B. Birge, PhD, New Jersey Medical School Cancer Center, Rutgers University, Newark, NJ, USA, Email: birgera@njms.rutgers.edu; Yi Wu, MD., PhD, Sol Sherry Thrombosis Research Center, Temple University School of Medicine, Philadelphia, PA, USA, Email: yiwu99@gmail.com.

Email address:

Liang Gao: evayaz@163.com

Chao He: echo_hc@163.com

Aizhen Yang: yangaizhen@suda.edu.cn

Haibin Zhou: zhouhaibin@suda.edu.cn

Qingxian Lu: q.lu@louisville.edu

Raymond B. Birge: birgera@njms.rutgers.edu

Yi Wu: yiwu99@gmail.com

Short title: Role of Tyro3, Axl, and Mertk in arthritis

# Abstract

Tyro3, Axl, and Mertk (abbreviated TAMs) comprise a family of homologous type 1 receptor tyrosine kinases (RTKs) that have been implicated as inhibitory receptors that dampen inflammation, but their roles in the pathogenesis of rheumatoid arthritis remains understudied. Here, to investigate TAMs in an inflammatory arthritis model, antibody-induced arthritis in single TAM-deficient mice (Tyro3- KO, Axl-KO, MertkKO) was induced by K/BxN serum injection. Subsequently, joint inflammation and cytokine levels, as well as the expression of FcγRs and complement receptors were assessed in WT and TAM-deficient mice. Compared with littermate control mice, Axl^/-^ and Mertk^-/-^ mice developed more severe antibody-induced arthritis, while in contrast, Tyro3^-/-^ mice showed diminished joint inflammation. Concomitantly, the levels of cytokines in joints of Axl^-/-^ and Mertk^-/-^ mice were also significantly increased, while cytokines in the Tyro3^-/-^ joint tissues were decreased. At the molecular and cellular level, TAMs showed distinct expression patterns, whereby monocytes expressed Axl and Mertk, but no Tyro3, while neutrophils expressed Axl and Tyro3 but little Mertk. Moreover, expression of Fcγ receptors and C5aR showed different patterns with TAMs expression, whereby FcγRIV was higher in monocytes of Axl^-/-^ and Mertk^-/-^ mice compared to wild-type mice, while Tyro3^-/-^ neutrophils showed lower expression levels of FcγRI, FcγRIII and FcγRIV. Finally, expression of C5aR was increased in Mertk^-/-^ monocytes, and was decreased in Tyro3^-/-^ neutrophils. These data indicate that Axl, Mertk and Tyro3 have distinct functions in antibody-induced arthritis, due in part to the differential regulation of cytokines production, as well as expression of FcγRs and C5aR.

**Keywords:** arthritis, immune complex, receptor tyrosine kinase, cytokine, Fcγ receptor **Introduction**

Rheumatoid arthritis (RA) is a common autoimmune disease that is characterized by progressive systemic inflammation and chronic joint damage^(1)^. Pathophysiologically, autoantibodies are detected in a majority of patients with RA, including rheumatoid factor, anti-citrullinated protein antibody (Ab), antibodies to Ig-binding protein, type II collagen, glucose-6-phosphate isomerase (GPI), a-enolase, and other antigens^(2-4)^. Autoantibody-positive patients with RA are characterized by a more severe disease course, higher cytokine levels, increased bone damage, and a lower potential for drugfree remission^(2, 4)^. Studies in murine models have demonstrated that autoantibodies against GPI, type II collagen, and fibrinogen induce arthritis^(5-7)^, suggesting that autoantibodies possess arthritogenic potential. Immune complexes formed by autoantibodies with their antigens may cause common effector mechanisms that contribute to disease development and progression^(8)^. Presently, the mechanisms underlying how the autoantibodies become arthritogenic remains poorly understood. Detailed characterization of the early innate immune response induced by autoantibodies should provide fundamental insights into the pathogenesis of RA and the onset of clinical symptoms.

Tyro3, Axl, and Mertk are three homologous type I receptor tyrosine kinase (RTK) that comprise the TAM family members ^(1)^. TAMs have similar domains and organization, with the extracellular domain containing two immunoglobulin-like and two fibronectin type III repeats in tandem, followed by a unilateral transmembrane domain and a cytoplasmic protein tyrosine kinase^(2, 3, 9)^. TAMs are mainly expressed on leukocytes such as macrophages, monocytes, and neutrophils, and are functionally complex, with important roles in hemostasis and inflammation as well as in proliferation, survival, cell adhesion and migration^(5, 10-13)^, inhibition of granulocytes adhesion to the endothelium^(14)^, and stabilization of blood clots^(15)^. Importantly, and with particular relevance in the context of RA, TAMs can also finely regulate the inflammatory cascade^(16)^ and mediate the engulfment of apoptotic corpses^(17)^, contributing to prevent the development of autoimmune reactions and inflammatory cascades. Consistent with this idea, Waterburg et al found that Axl and Mer have a protective role during joint inflammation^(18, 19)^. Similarly, the agonists of TAMs alleviate collagen-induced rheumatoid arthritis^(7)^. However, another study showed that Tyro3 supports arthritis^(20)^. Although these observations suggest differential involvement of TAMs in arthritis, the underlying mechanism remains enigma. In this study, we performed a comparison study of the phenotypes of single TAM gene deficient mice in the K/BxN serum transfer-induced arthritis (K/BxN-STIA) model and inveistigated the potential mechanism by which Tyro3, Axl, and Mertk have distinct or overlapping functions in

RA.

In this study, our results demonstrate that Tyro3, Axl, and Mertk have distinct functions in the pathogenesis of antibody-induced arthritis, Axl and Mertk are protective towards arthritic pathogenesis, while Tyro3 augments disease development. The distinct roles of TAM are associated with differential modulation of cytokines production in joint tissue, and different expression of Fc receptors (FcRs) and C5aR receptor (C5aR) on monocytes versus neutrophils. These findings not only uncover the distinct function of TAM in modulation of immune response, but also provide new insights into the pathogenesis of antibody-induced arthritis and identify potential therapeutic targets for the treatment of RA.

# RESULTS

**Axl or Mertk deficiency increases the severity of antibody-induced arthritis, but the deficiency of Tyro3 inhibits this process**.

To better understand the function of TAM receptors in the pathogenesis of autoantibody-induced arthritis, we examined the phenotype of knockout mice lacking Tyro3, Axl or Mertk in the K/BxN-STIA model. As shown in Figure 1(A-E), the WT control mice developed joint swelling and redness after receiving K/BxN serum injection, the mice lacking Axl or Mertk exhibited more severe joint swelling over the disease course. In contrast, Tyro3-deficient mice developed less joint swelling compared with their WT littermate control mice (Figure 1, A-E). The histological changes in joint inflammation of these knockout mice were evaluated by staining with hematoxylin and eosin, as well as Safranin O. The joints from WT mice that received K/BxN serum displayed inflamed and hyperplastic synovium with bone destruction, mononuclear cell infiltration, and pannus formation (Figure 2, A-C). Axl^-/-^ and Mertk^/-^ mice had more severe synovial inflammation, bone and cartilage destruction than their littermate control mice, but the Tyro3^-/-^ mice had significantly less joint inflammation than their littermates (Figure 2, D-F). Taken together, aforementioned results suggest that TAMs have distinct functions in the pathogenesis of antibody-induced arthritis; Axl and Mertk are protective against antibody-induced synovial inflammation, while

Tyro3 is permissive for K/BxN-mediated synovial pathology.

# The deficiency of Axl, Mertk and Tyro3 differentially alter the levels of cytokines in joint tissues of arthritic mice

Because levels of cytokines in joint tissue are consistent with severity of arthritis and clinical scores, we assessed cytokines levels of joint tissues on day 12 after injection of K/BxN serum. As shown in Figure 3A, the protein levels of cytokines TNF-α, IL-1β, IL-6, IL-17A, IP-10, IL-10 in joint homogenates of wild-type mice that received K/BxN serum transfer were significantly higher than the control mice without K/BxN serum injection. However, Axl^-/-^ mice had significantly higher levels of TNF-α, IL-1β, IL-6 and IL-17A than their littermate control mice (Figure 3A). Expression of these cytokines at the mRNA levels in the ankle joint tissue were measured by quantitative RT-PCR. The mRNA levels of TNF-α, IL-1β, IL-6 and IL-17A expression in Axl^-/-^ joint tissues of arthritic mice are consistent with their protein levels (Figure 3B). Expression of cytokines IL-17A, IP-10 and IL-12p70 in the joint tissues of Mertk^-/-^ mice were significant increased at the protein level, compared with those of wild-type mice (Figure 4, A). However, unlike Axl^-/-^ mice, Mertk^-/-^ mice had comparable levels of TNFα, IL-Iβ and IL6 with wild-type mice. The increased mRNA expression of IL17A, IP-10, IL-12p35, and IL-12p40 in the joint tissues of Mertk^-/-^ mice were consistent with their protein levels (Figure 4, A and B). In contrast to the mice lacking Axl or Mertk, Tyro3^-/-^ mice that received K/BxN serum had a significant reduction in IL-1β, IL-6, IP-10, and G-CSF in joints at mRNA expression (Figure 5). Taken together, the differential cytokine expression levels in mice lacking Axl, Mertk and Tyro3 are consistent with their phenotypes in arthritis, suggesting that TAMs differentially regulate antibody-induced inflammatory responses that contribute to the development

of arthritis.

**The deficiency of Axl and Mertk selectively upregulates the expression of FcγRIV in monocytes and the deficiency of Tyro3 down-regulates the expression of FcγRI,**

# FcγRIII, and FcγRIV in neutrophils

Previous studies have shown that monocytes and neutrophils contribute to the pathogenesis of arthritis. FcγRs, including FcγRI, FcγRIII and FcγRIV are antibody receptors on monocytes and neutrophils. FcγRIII and FcγRIV have been shown to promote the onset of arthritis in K/BxN-STIA model. We thus tested whether the contribution of TAM receptors to antibody-induced arthritis is related with the alternation of FcγRs expression on monocytes and neutrophils. As shown by RTPCR, the Axl mRNA was expressed in both monocytes and neutrophils, but Mertk was only expressed in monocytes and Tyro3 was only expressed in neutrophils (Figure 6, A and B). Both Axl deficient and Mertk deficient monocytes had significantly higher expression of FcγRIV than wild-type monocytes (Figure 6, C).

The expression of four FcγRs in Axl deficient neutrophils remained normal (Figure 6, D), the Tyro3 deficiency significantly decreased the expression of FcγRI, FcγRIII, and FcγRIV in neutrophils (Figure 6, D). These results suggest that differential roles of TAMs in the pathogenesis of antibody-induced arthritis may result from the differential modulation of FcγRs expression in monocytes or neutrophils.

# Axl, Mertk and Tyro3 differentially regulate the C5aR expression in leukocytes

In the K/BxN-STIA model, the arthritogenic Igs act through both Fc receptors and the complement C5a-C5aR, the alternative pathway of complement activation is critical for antibody-induced arthritis^(21)^. We tested whether TAMs modulate the expression of C5aR in monotypes and neutrophils. As shown by quantitative RT-PCR and flow cytometry, Mertk deficiency increased C5aR expression in monocytes, without affecting C3aR expression (Figure 7, A and B), Axl deficiency did not affect both C5aR and C3aR expression in monocytes (Figure 7, A and B). Tyro3 deficiency attenuated

C5aR in neutrophils (Figure 7C). All of TAM receptors did not affect the circulating levels of C3a and C5a in plasma of mice (Figure 8, A and B). Collectively, these data suggest that TAMs, by controlling the expression of C5aR levels, might regulate development of antibody-induced arthritis via a complement mediated pathway.

# Discussion

While TAM receptors have been implicated in various immune functions that include the regulation of efferocytosis, the suppression of inflammation, and pro-resolution, their role in regulation of innate immune cells affecting the development and severity of arthritis is not well understood. Here, in an effort to study TAMs function in the pathogenesis of antibody-induced arthritis, we investigated the phenotypes of the genetic knockouts of TAMs in K/BxN-STIA model. More specifically, we demonstrated that Axl or Mertk knockout enhances the severity of arthritis, while selective deficiency of Tyro3 in contrast attenuates arthritis. As such, these studies support the idea that pathophysiologically, Axl and Mertk suppress the pathogenesis of antibody-induced arthritis, while Tyro3 has a supportive role. These observations demonstrate that TAMs differentially participate in the pathogenesis of antibodyinduced arthritis, and contextually, may influence the future consideration of selective

TAMs agonists and therapeutics.

Historically, the capacities for TAMs to act as homeostatic receptors and dampen inflammatory responses in tissues was first demonstrated by the phenotype of Mertk kinase-dead (Mertk-KD) mice, characterized by an excessive production of TNF*α* upon LPS stimulation and death by endotoxic shock caused by sub-lethal doses of LPS^(22)^.

Subsequently, work by Lemke and colleagues showed that mutant mice lacking all three

TAMs (known as TAM^−/−^ mice) developed multiorgan symptoms typical of autoimmune inflammatory diseases^(23, 24)^. Phenotypically, TAM^−/−^ mice also became progressively blind and sterile and showed gradual enlargement of secondary lymphoid organs caused by an uncontrolled proliferation of B/T lymphocytes^(24)^; Immunologically, TAM^-/-^ mice displayed a range of serological and histological manifestations including immunoglobulin deposits in glomeruli, circulating autoantibodies, vasculitic skin lesions, alopecia, and swollen joints^(23, 24)^. Such observations supported an additive role for TAMs and that the TAM family members might have similar roles in regulation of inflammatory response. Here, we provide new evidence that TAMs can differentially regulate immune outcomes using K/BxN mice that spontaneously developed severe polyarthritis by age of 4–5 weeks. As such, a deficiency of Axl or Mertk up-regulated the expression of FcγRIV in monocytes, without affecting the expression of FcγRs in neutrophils. In contrast, a deficiency of

Tyro3 significantly down-regulated the expression of FcγRI, FcγRIII and FcγRIV in neutrophils. Furthermore, Mertk inhibited the expression of C5aR in monocytes, and

Tyro3 potentiates C5aR expression in neutrophils. Indeed, previous studies using

K/BxN-STIA model have shown that FcγRs have important functions, whereby FcγRI,

FcγRIII, and FcγRIV can promote the onset of arthritis, while FcγRIIB inhibits the onset of arthritis^(25-29)^. Clinical studies have also suggested that FcγRs are associated with the pathogenesis of rheumatoid arthritis^(30, 31)^. Our current studies suggest that the immune function of TAMs is dependent on the expression of FcγRs on the surface of monocytes or neutrophil membrane, by which Axl and Mertk inhibit the inflammatory reaction and attenuate the severity of arthritis, and Tyro3 promotes inflammation and increase the severity of arthritis. We hypothesize that observed distinct functions of the

TAM receptors in antibody-induced arthritis may result, at least in part, from the differential regulation of FcγRs expression and cytokines productions in monocytes versus neutrophils.

In addition to immune cells, cytokines also contribute an important role in the pathogenesis of arthritis. Previous studies have shown that cytokines, such as IL-1β, IL-6, TNF-α, IL-17A and IP-10 in ankle joints are significantly increased during the development of arthritis^(32-34)^, TNF-α and IL-1β may cause synovitis and bone and cartilage damage, then aggravating the incidence of arthritis^(35)^; IL-17A triggers changes in the synovium that lead to synovitis and maintain local inflammation. IL-6 and IL-17A can promote articular cartilage and bone damage^(36-39)^; the concentration of IP-10 and IL-12 is closely related to the pathogenesis of rheumatoid arthritis^(40, 41)^. Our present results indicate that Axl and Mertk inhibit articular cartilage and bone damage by down-regulation selective cytokines in joint tissues while Tyro3 has an opposing function to affect the onset of rheumatoid arthritis. While these post-receptor events are likely to be mechanistically complex and multi-factorial, the fact that Protein S (Pros1) appears to be a preferential ligand for Tyro3 could also imply differential regulation of immune arthritis by Gas6 and Pros1. Outside the scope of this current study, further studies comparing effects of Gas6 and Pros1 will be meritorious to better understand the dichotomy of TAMs function observed in this study as well as how TAMs ligands differentially regulate cytokine production from distinct leukocytes.

Finally, with respect to cell subtypes, monocytes and neutrophils also play a key role in the pathogenesis of arthritis. For example, antibody depletion studies and removal of monocytes or neutrophils from mice can inhibit the onset of arthritis^(42, 43)^. Furthermore, neutrophils change the permeability of blood vessels by producing inflammatory cytokines and degranulation reactions, facilitating autoantibodies and immune cells top enter the joint cavity, which can further secrete cytokines, matrix metalloproteinases, serine proteases, aggregate proteins and expedite joint damage. Although TAM receptors are able to regulate the innate immune response, it is unclear how Axl, Mertk, and Tyro3 regulate innate immune cells affecting the severity of arthritis. Based on the current data and knowledge, the functional difference among TAMs was perhaps due to the distinct patterns of TAMs expression in monocytes and neutrophils, Fc receptors expression, and C5aR expression. All of these proteins are key players in the pathogenesis of arthritis^(21)^. Our future work will focus on the dissection of the connections between the TAM receptors and the expression of cytokines in both monoctyes and neutrophils. Previous studies on the role of TAM in inherited photoreceptor degenerations using the knockout mice suggest the association of the of Mertk and Tyro-3 function in retinal pigment epithelium with C57BL/6 (B6) allele and 129 allele^(44)^, and the loss of Mertk might affects the expression of Tyro-3^(45)^, whether the function of each TAM receptor is associated the modifier allele and the altered the expression of the paralog in leukocytes await further investigation.

In conclusion, this study demonstrates the distinct immunological roles of the TAM receptors in the pathogenesis of antibody-induced arthritis. This new finding not only advances our understanding of the pathogenesis of rheumatoid arthritis, but may ultimately helps reveal new therapeutic target for the treatment of rheumatoid arthritis.

**Materials and Methods**

# Knockout mice and generation of K/BxN serum

The knockout mice of Mertk, Axl or Tyro3 were characterized in our previous study^(46)^.

Mertk-KO mice and their control Mertk-WT mice were in SV129 background. Tyro3-

KO mice with their control Tyro3-WT mice, and Axl-KO mice with their control AxlWT mice have been backcrossed in C57BL/6 background for more than ten generations. For each type of KOs, the heterozygous mice were intercrossed to generate homozygous null offspring and the littermate controls, which were used in the experiments. Mice were fed standard rodent chow and water ad libitum, and were maintained under climate-controlled conditions in a pathogen-free facility in a 12-h light/dark cycle. The animal protocol was approved by the Institutional Animal Care and Use Committee (IACUC) of Soochow University and the health status of the animals was monitored in accordance with the guidelines of the IACUC. K/BxN T cell receptor transgenic mice are a model of inflammatory arthritis, similar to rheumatoid arthritis. Disease in these animals is focused specifically on the joints but stems from production of antibody recognizing a ubiquitously expressed antigen, glucose-6phosphate isomerase (GPI). The male NOD/ShiLtJ mice that carried major histocompatibility class II molecule Ag7 (The Jackson Laboratory, Bar Harbor, ME,

USA) were crossed with the female KRN T-cell receptor–transgenic mice to generate K/BxN mice. As we previously described, after the K/BxN mice developed severe polyarthritis spontaneously by age of 4–5 week, their serum (KBxN serum) was

prepared^(47)^.

# Induction of K/BxN-STIA model

Transfer of K/BxN serum into disease-free mice induced a similar arthritis receipt mice because of the delivery of autoantibodies recognizing GPI in K/BxN serum. Tyro3, Axl and Mertk-knockout mice and their littermate control WT mice, at 8-week-old with body weight between 20 and 24 g, received intraperitoneal injections of 150 μL of K/BxN serum on day 0 and 2. As we previously described, ankle joint diameter was measured everay day using calipers^(48)^, and the limbs of the mice were clinically scored indicating the severity of the joints of the mice. In brief, clinical score (total = 12) for 4

paws was used for the measurement of disease severity and was scoredas follows:0=normal, 1=swollenwrist/ankle, 2 = swelling extending to forepaw/hind paw, and 3 =swelling extending to digits.

# Histological analysis

At the end of each experiment, the mice were euthanized by CO_2_ narcosis**.** Hind limb joints were removed and fixed in the buffered formalin. After the samples were decalcified in formic acid (Fisher Scientific), they were embedded in paraffin. After removal of the paraffin, the sections were stained with hematoxylin and eosin (H&E) and Safranin O. The sections were photographed by a confocal laser-scanning microscope (Leica TCS SP5). The images were processed using Adobe Photoshop 9.0 software. As previously described, the severity of arthritis was examined by grading the cellular infiltration and joint destruction as follows: 0, normal; 1, minimal; 2, mild; 3, moderate; 4, marked; 5, severe ^(49)^.

# Measurement of cytokines in joint tissues

The ankle joint were collected and homogenized in 300 μL PBS containing protease inhibitor (Complete Protease Inhibitor Cocktail, Roche). The samples were centrifuged at 14,000 rpm for 30 minutes to remove insoluble precipitates, and the supernatant was collected. The cytokines levels were measured using the MILLIPLEX Kit (Millipore). Briefly, the samples were incubated with the beads that were coated with specific capture antibody overnight at 4°C. Incubation of biotinylated detection antibodies and Streptavidin-PE conjugate with the samples and the subsequent measurement were performed according to the manufacturer’s instruction. To verify the data, analysis of cytokine gene expression was also performed with quantitative RT-PCR.

# Isolation of monocytes and neutrophils from mice

As previously described we purified monocytes and neutrophils from control diseasefree and arthritis mice^(50)^. In brief, mouse monocytes are defined as CD11b^high^ (CD90/B220/CD49b/NK1.1/Ly6G/Ter-119) ^low^. To purify monocytes, peripheral blood was collected by cardiac puncture using 3.8% sodium citrate as anticoagulant. Whole blood was incubated with a mixture of PE–conjugated antibodies against B220, CD49b, NK1.1, Ly-6G, CD90 and Ter-119, followed by lineage depletion using anti-PE magnetic beads (Miltenyi). To analyze immunophenotype, the antibodies used include anti-CD49bPE, anti CD49b-FITC, anti-CD90-PE, anti-CD90-FITC, anti-B220-PE, anti B220-FITC, anti-NK1.1-PE, anti-NK1.1-FITC, anti-CD11b-APC (BD Biosciences); anti-CD11b-PE (ED8) (Abcam), anti-CD11b-APC-Cy7 M1/70 (BD Biosciences), antiLy-6G-PE, anti-Ly-6G-FITC, anti-Ly-6C-FITC, anti-F4/80-biotin, anti-F4/80-FITC,

C1:A3-1 (BioLegend); anti-CD11c-biotin, anti-CD11c-FITC, anti-CD11c-APC, HL3 (BD Biosciences).

To putify neutrophils, bone marrow was harvested from femurs and tibias of mice and collected in RPMI 1640 supplemented with 10% FBS and 2 mM EDTA. Cells were filtered through 30-μm filter to remove cell clumps, and the cell suspension was centrifuged at 400 g for 10 min and resuspended in PBS containing 0.5% FBS and 2 mM EDTA. Neutrophils were separated by negative selection using the MACS magnetic bead separation system (Miltenyi Biotec) according to the manufacturer's instructions. Briefly, the resuspended cells were incubated with a cocktail of biotinconjugated monoclonal antibodies against antigens that are not expressed on neutrophils for 10 min at 4º C. Cells were then loaded onto MS columns connected to MACS Separator. The eluted neutrophils were identified by determining CD11b and

Ly6G positive using flow cytometry.

# Measurement of mRNA expression by reverse-transcription polymerase chain reaction (RT-PCR) and quantitative real time RT-PCR (qPCR)

Total RNA was isolate from joint tissue or cells using Qiagen RNeasy minikits, and was used as template in a one-step RT-PCR reaction (SuperScript One-Step RT-PCR with Platinum^®^ *Taq*, Invitrogen). RT for cDNA synthesis was performed in incubation at 50°C for 30 minutes, followed by PCR cycling as follows: initial denaturation at 94°C for 2 minutes followed by 30 cycles of 94°C for 15 seconds, annealing at 55°C for 30 seconds, and extension at 72°C for 1 minute. The concentration of the primers was 0.2 µmol/L. The PCR products were visualized in 4% agarose gel electrophoresis. As we previously described^(50)^, quantitative real-time PCR was performed by the Maxima SYBR Green/ROX quantitative PCR Master Mix kit (Fermentas) with an ABI 7500 system (Applied Biosystems) according to the manufacturer’s instructions. Target gene expression was normalized over the level of β-actin, and the results were analyzed by using ABI 7500 SDS Version 2.4 software. The primer sequences were listed in Table

1.

# Measurement of C3 and C5 levels in plasma

The levels of C3 and C5 in mouse plasma was measured using ELISA (R&D system) according to the manufacturer’s protocol.

# Statistical Analysis

In vitro and in vivo data are expressed as the mean ± SEM of at least three independent experiments, unless otherwise indicated. Data were analyzed using the statistical software GraphPad Prism 8. For parametric comparison, two-way ANOVA (analysis of variance) for multiple groups and the two-tailed Student’s t-test for 2 groups were used. Non-parametric comparison was performed by Mann Whitney U test. P values of

< 0.05 were considered statistically significant.

# Conclusions

This study demonstrates the distinct immunological roles of the TAM receptors in the pathogenesis of antibody-induced arthritis. This new finding not only advances our understanding of the pathogenesis of rheumatoid arthritis, but may ultimately helps reveal new therapeutic target for the treatment of rheumatoid arthritis. Our future work will focus on the dissection of the connections between the TAM receptors and the expression of cytokines.

# Abbreviations

TAM: Tyro3, Axl, Mertk; RTKs: receptor tyrosine kinases; FcRs: Fc receptors; RA: rheumatoid arthritis; C5aR: C5aR receptor; GPI: glucose-6-phosphate isomerase; Gas6:

Growth arrest-specific 6; Pros1: Protein S.

**Declarations:**

Ethical Approval and Consent to participate: This study was approved by the Medical

Ethics Committee of Soochow University.

Consent for publication: Not applicable.

Availability of supporting data：All data generated or analysed during this study are included and will be made available from the corresponding author in a reasonable request.

Competing interests: The authors declare that they have no competing interests.

Funding: This research was supported by grants from the National Natural Science

Foundation of China (81970128, 82170129, 82270136, 81301534, 31970890), by the Translational Research Grant of NCRCH (2020ZKPA02, 2020WSA04), the

collaboration fund from State Key Laboratory of Radiation Medicine and Protection

(GZN1201802), the Priority Academic Program Development of Jiangsu Higher

Education Institutions (PAPD).

Authors' contributions: L.G., C.H., A.Y. and H.Z. performed research, and collected and analyzed data. Q.L. provided knockout mice. R.B.B. conceived the study and helped the interpretation of the data and manuscript editing. A.Y. and Y.W. conceived the study and designed the experiments, supervised the research, analyzed the data and wrote the manuscript.

Acknowledgements：Not applicable.

Authors' information: Liang Gao: evayaz@163.com; Chao He: echo_hc@163.com;

Aizhen Yang: yangaizhen@suda.edu.cn; Haibin Zhou: zhouhaibin@suda.edu.cn; Qingxian Lu: q.lu@louisville.edu; Raymond B. Birge: birgera@njms.rutgers.edu; Yi

Wu: yiwu99@gmail.com

# References

1. Lemke G, Rothlin CV. Immunobiology of the TAM receptors. Nat Rev Immunol. 2008;8(5):327-36.
2. Hilliard BA, Zizzo G, Ulas M, Linan MK, Schreiter J, Cohen PL. Increased expression of Mer tyrosine kinase in circulating dendritic cells and monocytes of lupus patients: correlations with plasma interferon activity and steroid therapy.

Arthritis Res Ther. 2014;16(2):R76.

1. Zhu H, Sun X, Zhu L, Hu F, Shi L, Fan C, et al. Different expression patterns and clinical significance of mAxl and sAxl in systemic lupus erythematosus. Lupus.

2014;23(7):624-34.

1. Lew ED, Oh J, Burrola PG, Lax I, Zagórska A, Través PG, et al. Differential TAM receptor-ligand-phospholipid interactions delimit differential TAM bioactivities.

Elife. 2014;3.

1. Tsou WI, Nguyen KQ, Calarese DA, Garforth SJ, Antes AL, Smirnov SV, et al. Receptor tyrosine kinases, TYRO3, AXL, and MER, demonstrate distinct patterns and complex regulation of ligand-induced activation. J Biol Chem. 2014;289(37):2575063.
2. Zagórska A, Través PG, Lew ED, Dransfield I, Lemke G. Diversification of TAM receptor tyrosine kinase function. Nat Immunol. 2014;15(10):920-8.
3. van den Brand BT, Abdollahi-Roodsaz S, Vermeij EA, Bennink MB, Arntz OJ,

Rothlin CV, et al. Therapeutic efficacy of Tyro3, Axl, and Mer tyrosine kinase agonists in collagen-induced arthritis. Arthritis Rheum. 2013;65(3):671-80.

1. Nakamura YS, Hakeda Y, Takakura N, Kameda T, Hamaguchi I, Miyamoto T, et al. Tyro 3 receptor tyrosine kinase and its ligand, Gas6, stimulate the function of osteoclasts. Stem Cells. 1998;16(3):229-38.
2. Scutera S, Fraone T, Musso T, Cappello P, Rossi S, Pierobon D, et al. Survival and migration of human dendritic cells are regulated by an IFN-alpha-inducible Axl/Gas6 pathway. J Immunol. 2009;183(5):3004-13.
3. Lew ED, Oh J, Burrola PG, Lax I, Zagorska A, Traves PG, et al. Differential TAM receptor-ligand-phospholipid interactions delimit differential TAM bioactivities.

Elife. 2014;3.

1. Zagorska A, Traves PG, Lew ED, Dransfield I, Lemke G. Diversification of TAM receptor tyrosine kinase function. Nat Immunol. 2014;15(10):920-8.
2. Goruppi S, Ruaro E, Schneider C. Gas6, the ligand of Axl tyrosine kinase receptor, has mitogenic and survival activities for serum starved NIH3T3 fibroblasts.

Oncogene. 1996;12(3):471-80.

1. Sainaghi PP, Castello L, Bergamasco L, Galletti M, Bellosta P, Avanzi GC. Gas6 induces proliferation in prostate carcinoma cell lines expressing the Axl receptor. J Cell Physiol. 2005;204(1):36-44.
2. Avanzi GC, Gallicchio M, Bottarel F, Gammaitoni L, Cavalloni G, Buonfiglio D, et al. GAS6 inhibits granulocyte adhesion to endothelial cells. Blood.

1998;91(7):2334-40.

1. Angelillo-Scherrer A, Burnier L, Flores N, Savi P, DeMol M, Schaeffer P, et al. Role of Gas6 receptors in platelet signaling during thrombus stabilization and implications for antithrombotic therapy. J Clin Invest. 2005;115(2):237-46.
2. Alciato F, Sainaghi PP, Sola D, Castello L, Avanzi GC. TNF-alpha, IL-6, and IL1 expression is inhibited by GAS6 in monocytes/macrophages. J Leukoc Biol.

2010;87(5):869-75.

1. Anderson HA, Maylock CA, Williams JA, Paweletz CP, Shu H, Shacter E. Serum-derived protein S binds to phosphatidylserine and stimulates the phagocytosis of apoptotic cells. Nat Immunol. 2003;4(1):87-91.
2. Waterborg CEJ, Broeren MGA, Blaney Davidson EN, Koenders MI, van Lent P, van den Berg WB, et al. The level of synovial AXL expression determines the outcome of inflammatory arthritis, possibly depending on the upstream role of TGFβ1. Rheumatology (Oxford). 2019;58(3):536-46.
3. Waterborg CEJ, Beermann S, Broeren MGA, Bennink MB, Koenders MI, van Lent P, et al. Protective Role of the MER Tyrosine Kinase via Efferocytosis in Rheumatoid Arthritis Models. Front Immunol. 2018;9:742.
4. Ruiz-Heiland G, Zhao Y, Derer A, Braun T, Engelke K, Neumann E, et al. Deletion of the receptor tyrosine kinase Tyro3 inhibits synovial hyperplasia and bone damage in arthritis. Ann Rheum Dis. 2014;73(4):771-9.
5. Ji H, Ohmura K, Mahmood U, Lee DM, Hofhuis FM, Boackle SA, et al. Arthritis critically dependent on innate immune system players. Immunity. 2002;16(2):15768.
6. Camenisch TD, Koller BH, Earp HS, Matsushima GK. A novel receptor tyrosine kinase, Mer, inhibits TNF-alpha production and lipopolysaccharide-induced endotoxic shock. J Immunol. 1999;162(6):3498-503.
7. Lu Q, Gore M, Zhang Q, Camenisch T, Boast S, Casagranda F, et al. Tyro-3 family receptors are essential regulators of mammalian spermatogenesis. Nature.

1999;398(6729):723-8.

1. Lu Q, Lemke G. Homeostatic regulation of the immune system by receptor tyrosine kinases of the Tyro 3 family. Science. 2001;293(5528):306-11.
2. Corr M, Crain B. The role of FcgammaR signaling in the K/B x N serum transfer model of arthritis. J Immunol. 2002;169(11):6604-9.
3. van Lent PL, Grevers L, Lubberts E, de Vries TJ, Nabbe KC, Verbeek S, et al. Fcgamma receptors directly mediate cartilage, but not bone, destruction in murine antigen-induced arthritis: uncoupling of cartilage damage from bone erosion and joint inflammation. Arthritis Rheum. 2006;54(12):3868-77.
4. Nimmerjahn F, Lux A, Albert H, Woigk M, Lehmann C, Dudziak D, et al.

FcgammaRIV deletion reveals its central role for IgG2a and IgG2b activity in vivo.

Proc Natl Acad Sci U S A. 2010;107(45):19396-401.

1. Mancardi DA, Jonsson F, Iannascoli B, Khun H, Van Rooijen N, Huerre M, et al. Cutting Edge: The murine high-affinity IgG receptor FcgammaRIV is sufficient for autoantibody-induced arthritis. J Immunol. 2011;186(4):1899-903.
2. Hobday PM, Auger JL, Schuneman GR, Haasken S, Verbeek JS, Binstadt BA.

Fcgamma receptor III and Fcgamma receptor IV on macrophages drive autoimmune valvular carditis in mice. Arthritis Rheumatol. 2014;66(4):852-62.

1. Tsuboi N, Ernandez T, Li X, Nishi H, Cullere X, Mekala D, et al. Regulation of human neutrophil Fcgamma receptor IIa by C5a receptor promotes inflammatory arthritis in mice. Arthritis Rheum. 2011;63(2):467-78.
2. Blom AB, Radstake TR, Holthuysen AE, Sloetjes AW, Pesman GJ, Sweep FG, et al. Increased expression of Fcgamma receptors II and III on macrophages of rheumatoid arthritis patients results in higher production of tumor necrosis factor alpha and matrix metalloproteinase. Arthritis Rheum. 2003;48(4):1002-14.
3. Smolen JS, Aletaha D, McInnes IB. Rheumatoid arthritis. The Lancet.

2016;388(10055):2023-38.

1. Iwanami K, Matsumoto I, Tanaka-Watanabe Y, Inoue A, Mihara M, Ohsugi Y, et al. Crucial role of the interleukin-6/interleukin-17 cytokine axis in the induction of arthritis by glucose-6-phosphate isomerase. Arthritis Rheum. 2008;58(3):754-63. 34. Mikuls TR, Moreland LW. TNF blockade in the treatment of rheumatoid arthritis: infliximab versus etanercept. Expert Opin Pharmacother. 2001;2(1):75-84.
2. Ji H, Pettit A, Ohmura K, Ortiz-Lopez A, Duchatelle V, Degott C, et al. Critical roles for interleukin 1 and tumor necrosis factor alpha in antibody-induced arthritis. J Exp Med. 2002;196(1):77-85.
3. Jacobs JP, Wu HJ, Benoist C, Mathis D. IL-17-producing T cells can augment autoantibody-induced arthritis. Proc Natl Acad Sci U S A. 2009;106(51):21789-94. 37. Sadik CD, Kim ND, Alekseeva E, Luster AD. IL-17RA signaling amplifies antibody-induced arthritis. PLoS One. 2011;6(10):e26342.
4. Lubberts E, Joosten LA, Oppers B, van den Bersselaar L, Coenen-de Roo CJ, Kolls JK, et al. IL-1-independent role of IL-17 in synovial inflammation and joint destruction during collagen-induced arthritis. J Immunol. 2001;167(2):1004-13.
5. Chabaud M, Lubberts E, Joosten L, van Den Berg W, Miossec P. IL-17 derived from juxta-articular bone and synovium contributes to joint degradation in rheumatoid arthritis. Arthritis Res. 2001;3(3):168-77.
6. van Hooij A, Boeters DM, Tjon Kon Fat EM, van den Eeden SJF, Corstjens P, van der Helm-van Mil AHM, et al. Longitudinal IP-10 Serum Levels Are Associated with the Course of Disease Activity and Remission in Patients with Rheumatoid Arthritis. Clin Vaccine Immunol. 2017;24(8).
7. Paradowska-Gorycka A, Sowinska A, Stypinska B, Haladyj E, Pawlik A,

Romanowska-Prochnicka K, et al. IL-12B Gene Polymorphisms and IL-12 p70 Serum Levels Among Patients with Rheumatoid Arthritis. Scand J Immunol. 2017;85(2):14754.

1. Misharin AV, Cuda CM, Saber R, Turner JD, Gierut AK, Haines GK, 3rd, et al. Nonclassical Ly6C(-) monocytes drive the development of inflammatory arthritis in mice. Cell Rep. 2014;9(2):591-604.
2. Wipke BT, Allen PM. Essential role of neutrophils in the initiation and progression of a murine model of rheumatoid arthritis. J Immunol.

2001;167(3):1601-8.

1. Vollrath D, Yasumura D, Benchorin G, Matthes MT, Feng W, Nguyen NM, et al. Tyro3 Modulates Mertk-Associated Retinal Degeneration. PLOS Genetics.

2015;11(12):e1005723.

1. Akalu YT, Mercau ME, Ansems M, Hughes LD, Nevin J, Alberto EJ, et al. Tissue-specific modifier alleles determine Mertk loss-of-function traits. eLife.

2022;11:e80530.

1. Zhou J, Yang A, Wang Y, Chen F, Zhao Z, Davra V, et al. Tyro3, Axl, and Mertk receptors differentially participate in platelet activation and thrombus formation. Cell Commun Signal. 2018;16(1):98.
2. Yang A, Zhou J, Wang B, Dai J, Colman RW, Song W, et al. A critical role for plasma kallikrein in the pathogenesis of autoantibody-induced arthritis. Faseb j.

2017;31(12):5419-31.

1. Xie Z, Dai J, Yang A, Wu Y. A role for bradykinin in the development of anticollagen antibody-induced arthritis. Rheumatology (Oxford). 2014;53(7):1301-6.
2. Kim ND, Chou RC, Seung E, Tager AM, Luster AD. A unique requirement for the leukotriene B4 receptor BLT1 for neutrophil recruitment in inflammatory arthritis.

J Exp Med. 2006;203(4):829-35.

50. Yang A, Xie Z, Wang B, Colman RW, Dai J, Wu Y. An essential role of highmolecular-weight kininogen in endotoxemia. J Exp Med. 2017;214(9):2649-70.

# Figure legends

**Figure 1. Mice deficient of Axl or Mertk have a significant increase in antibodyinduced arthritis, but Tyro3 deificent mice have an opposite phenotype.** Axl^-/-^ mice, Mertk^-/-^ mice or Tyro3^-/-^ mice and their littermate WT controls received intraperitoneal injection of 150 μL of K/BxN serum twice on d 0 and d 2. Joint diameter (A) and clinical scores (B) was evaluated every day, and the changes from that measured on d 0 were recorded. The hind paws of Axl^-/-^ mice (C) , Mertk^-/-^ mice (D), Tyro3^-/-^ mice (E) and their littermate WT controls were photographed on day 0 and day 8 after K/BxN serum injection. Data are means ± SEM, n=8. Joint diameters were abalyzed using Unpaired t test with Welch's correction (A). Clinical scores were anaylzyed by Mann

Whitney U test (B). *, P<0.05; **, P<0.01.

**Figure 2. Deficiency of Axl or Mertk enhances antibody-induced arthritis**, **but**

# Tyro3 deficiency has an inhibitory effect. Arthritis in the knockout mice lacking

Tyro3, Axl or Mertk and their littermate control was induced by K/BxN serum injection. On day 12 the mice were euthanized, and the hind ankle joints were removed. Joints were fixed in 4% paraformaldehyde and decalcified. Paraffin-embedded sections were stained with hematoxylin and eosin (H&E) and Safranin O and photographed under a microscope. Representative histologic images of the staining with H&E staining and Safranin O staining are shown(A-C). Histological score for bone erosion, cartilage erosion and synovitis between Tyro3-, Axl-, or Mertk-knockout mice and their littermate controls was calculated and compared (D-E), n=6. Mann Whitney U test. *, p<0.05; **, p<0.01. Ca, calcaneus; S, synovium; Ta, talus; Ti, tibia; M, bone marrow; C, cartilage.

**Figure 3. Deficiency of Axl increases cytokine expression in joint tissue of mice bearing arthritis.** The knockout mice lacking Axl and their littermate control received K/BxN serum injection. On day 12 the mice were euthanized, followed by removal of the ankle joints. (A) Cytokine levels in joint homogenates were measured as described in the Methods. (B) RNA was purified from joint tissue and cytokine mRNA levels were measured by quantitative RT-PCR. The results were normalized against mRNA of β-actin and relative levels of quantification were calculated. Data are means **±** SEM, n=6. For comparison of the effect of gene deficiency, two way ANOVA with the

Tukey’s post hoc correction analyses was used. *, p<0.05; **, p<0.01.

**Figure 4. Mertk deficiency increases cytokine expression in joint tissue of mice bearing arthritis.** The knockout mice lacking Mertk and their littermate control received K/BxN serum injection. On day 12 the mice were euthanized, followed by removal of the ankle joints. (A) Cytokine levels in joint homogenates were measured as described in the Methods. (B) RNA was purified from joint tissue and cytokine mRNA levels were measured by quantitative RT-PCR. The results were normalized against mRNA of β-actin and relative levels of quantification were calculated. Data are means **±** SEM, n=6. For comparison of the effect of gene deficiency, two way ANOVA with the Tukey’s post hoc correction analyses was used. *, p<0.05; **, p<0.01.

**Figure 5. Tyro3 deficiency decreases cytokine expression in joint tissue of mice bearing arthritis.** The knockout mice lacking Tyro3 and their littermate control received K/BxN serum injection. On day 12 the mice were euthanized, followed by removal of the ankle joints. RNA was purified from joint tissue and cytokine mRNA levels were measured by quantitative RT-PCR. The results were normalized against mRNA of β-actin and relative levels of quantification were calculated. Data are means **±** SEM, n=6. For comparison of the effect of gene deficiency, two way ANOVA with the Tukey’s post hoc correction analyses was used. *, p<0.05; **, p<0.01.

**Figure 6. Axl and Mertk suppress FcγRIV expression in monocytes and Tyro3 enhances the expression of FcγRI, FcγRIII and FcγRIV in neutrophils.** Expression of Axl, Mertk and Tyro3 at mRNA levels in monocytes and neutrophils were measured by RT-PCR(A, B). Expression of FcγRS on monocytes and neutrophils from Tyro3^-/-^, Axl^-/-^ or Mertk^-/-^ mice and their control mice were measured by qPCR (C, D). n=6. Data are means **±** SEM. Student’s t test. *, p<0.05; **, p<0.01; ***, p<0.001.

**Figure 7. Mertk deficiency increases expression of C5aR in monocytes, Tyro3 deficiency inhibits expression of C5aR in neutrophils.** Monocytes from Axl or Mertk deficient mice and control mice were isolated, the expression of C3aR and C5aR was measured by qPCR (A, B). Neutrophils from Tyro3 deficient mice and control mice were isolated, the expression of C5aR was measured by qPCR (C). Data are means **±**

SEM, n=6. Student’s t test. **, p<0.01.

# Figure 8. The deficiency of Axl, Mertk, or Tyro3 does not affect the levels of C3

**and C5 in plasma.** The levels of C3 (A) and C5 (B) in plasma from Tyro3^-/-^, Axl^-/-^ or Mertk^-/-^ and control mice were measured by ELISA. Data are means ± SEM, n=6.

Student’s t test.
