# Supplementary material for: Receptor tyrosine kinases Tyro3, Axl, and Mertk differentially contribute to antibody-induced arthritis
Source: Cell Commun Signal. 2023 Aug 3;21:195. doi: 10.1186/s12964-023-01133-0 (PMC10398921; doi:10.1186/s12964-023-01133-0)
Supplement: Supplementary file 3 — Additional file 2. [file 12964_2023_1133_MOESM2_ESM.pptx]

## Slide 1
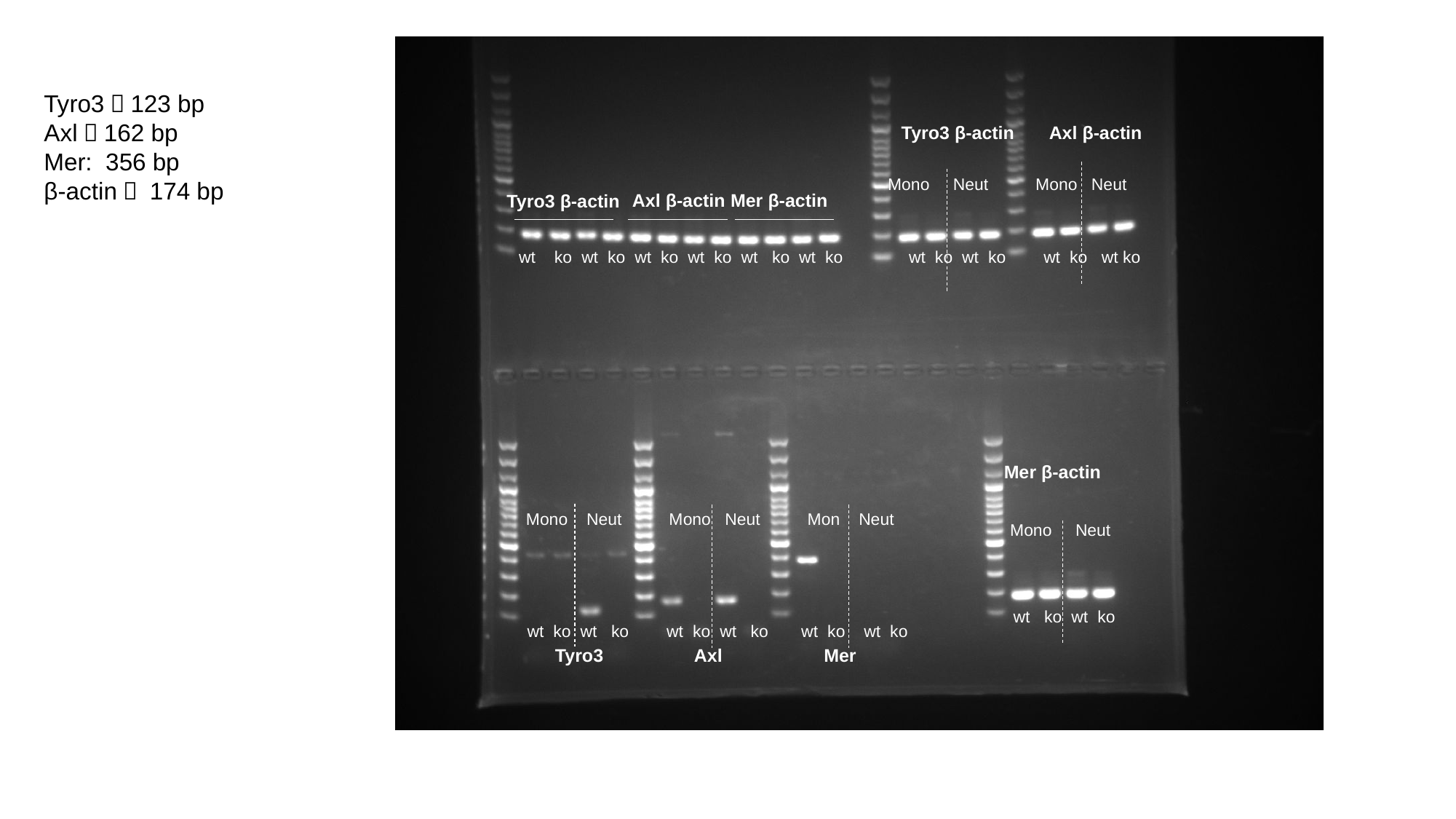

Tyro3 β-actin Axl β-actin
 Mono Neut Mono Neut Mon Neut
 wt ko wt ko wt ko wt ko wt ko wt ko
Tyro3 Axl Mer
wt ko wt ko wt ko wt ko wt ko wt ko wt ko wt ko wt ko wt ko
Mer β-actin
 wt ko wt ko
 Mono Neut Mono Neut
 Axl β-actin
Mer β-actin
Tyro3 β-actin
 Mono Neut
Tyro3：123 bp
Axl：162 bp
Mer: 356 bp
β-actin： 174 bp
